# Multivalent Immune-Protective Effects of Egg Yolk Immunoglobulin Y (IgY) Derived from Live or Inactivated *Shewanella xiamenensis* Against Major Aquaculture Pathogens

**DOI:** 10.3390/ijms26147012

**Published:** 2025-07-21

**Authors:** Jing Chen, Pan Cui, Huihui Xiao, Xiaohui Han, Ziye Ma, Xiaoqing Wu, Juan Lu, Guoping Zhu, Yong Liu, Xiang Liu

**Affiliations:** 1 Anhui Province Key Laboratory of Embryo Development and Reproductive Regulation, Fuyang Normal University, Fuyang 236041, China; 23211308@stu.fynu.edu.cn (J.C.); 23211320@stu.fynu.edu.cn (P.C.); 22211302@stu.fynu.edu.cn (H.X.); 2021114851@stu.fynu.edu.cn (X.H.); 2021114643@stu.fynu.edu.cn (Z.M.); 201806024@fynu.edu.cn (X.W.); 2 Anhui Provincial Key Laboratory of Molecular Enzymology and Mechanism of Major Metabolic Diseases, Auhui Provincial Engineering Research Centre for Molecular Detection and Diagnostics, College of Life Sciences, Anhui Normal University, Wuhu 241000, China; gpz2012@ahnu.edu.cn; 3 Rural Revitalization Collaborative Technology Service Center of Anhui Province, Fuyang Normal University, Fuyang 236041, China; 200107024@fynu.edu.cn

**Keywords:** IgY antibody, multivalent vaccine, *Shewanella xiamenensis*, *Aeromonas hydrophila*, passive immunity

## Abstract

Egg yolk immunoglobulin Y (IgY) possesses advantages such as low cost, easy availability, simple preparation, high antigen specificity, absence of drug residues, and compliance with animal welfare standards, making it an environmentally friendly and safe alternative to antibiotics. This research utilizes IgY antibody technology to develop a multivalent passive immune vaccine for major pathogenic bacteria in aquaculture. In this study, IgY antibodies against live *Shewanella xiamenensis* (LSX-IgY) and inactivated *S. xiamenensis* (ISX-IgY) were prepared by immunizing laying hens, and passive immunization protection experiments were conducted in *Carassius auratus* infected with *S. xiamenensis* and *Aeromonas hydrophila*. The passive immunization protection rates of LSX-IgY and ISX-IgY against *S. xiamenensis* were 63.64% and 72.73%, respectively, and the passive cross-protection rates against *A. hydrophila* were 50% and 71.43%, respectively. Further, *C. auratus* sera could specifically bind to *S. xiamenensis* or *A. hydrophila* in vitro, and the phagocytic activity of leukocytes was increased. LSX-IgY and ISX-IgY could reduce the bacterial load in the *C. auratus* kidneys. Meanwhile, they could significantly reduce the levels of antioxidant factors in serum and inhibit the mRNA expression of inflammation-related factors in the kidneys and spleens. Additionally, histopathology and immunofluorescence analysis showed that both IgY preparations preserved tissue integrity and reduced the expression of apoptosis factor (p53) and DNA damage factor (γH2A.X) of visceral organs, respectively. In summary, LSX-IgY and ISX-IgY can combat various bacterial infections, with no significant difference between the two. Additionally, inactivated bacterial immunization is more aligned with animal welfare standards for laying hens. Therefore, ISX-IgY is expected to serve as a multivalent vaccine against major aquaculture pathogens.

## 1. Introduction

Aquaculture is a globally significant food production industry, providing over 50% of the fish resources for human consumption [1]. However, with the expansion of farming, bacterial diseases have become a major bottleneck restricting the development of aquaculture [2,3]. In recent years, diseases caused by various pathogenic bacteria, such as *Aeromonas hydrophila*, *Shewanella xiamenensis*, *Aeromonas veronii*, *Vibrio vulnificus*, *Pseudomonas fluorescens*, and *Vibrio parahaemolyticus,* have frequently broken out, leading to large-scale deaths of farmed animals and significant economic losses [4]. Aquaculture is facing a dual threat from *S. xiamenensis* and *A. hydrophila*. *S. xiamenensis* is primarily distributed in aquaculture areas of Southeast Asia, exhibiting multidrug resistance and capable of causing highly fatal septicemia in fish and shrimp [5]. *A. hydrophila* is widely present in freshwater environments globally, capable of inducing hemorrhagic disease in fish, and its drug resistance is continuously increasing [6,7,8,9]. Additionally, both pathogens of *S. xiamenensis* and *A. hydrophila* pose zoonotic risks, threatening public health [10,11].

With the development of the aquaculture industry, severe challenges are encountered in the prevention and control of pathogenic bacteria. Among current control measures, traditional antibiotics (such as enrofloxacin and florfenicol) have limited efficacy due to resistance issues [12]. Probiotics (such as *Bacillus subtilis* and *Lactobacillus*) and traditional Chinese medicine preparations (such as extracts of *Rheum officinale* and *Scutellaria baicalensis*) can improve the microecological environment, but their effectiveness in preventing and controlling sudden infections is limited [13], while novel biological control technologies (such as bacteriophages and antimicrobial peptides) are still in the research and development stage [14]. Although existing vaccines have achieved success in controlling some pathogens, specific vaccines for emerging multidrug-resistant pathogens such as *S. xiamenensis* remain unavailable [3]. Particularly, *S. xiamenensis* has demonstrated significant resistance to existing prevention and control measures due to its unique drug resistance mechanisms and pathogenic characteristics, necessitating the urgent development of highly effective and environmentally friendly new vaccines.

There is a trend of diversified development in new vaccine technologies for aquaculture, mainly including four major technical approaches: genetically engineered subunit vaccines, which are designed with targeted precision by identifying pathogen antigen epitopes [15]; DNA vaccines, which achieve sustained in vivo expression by delivering antigen genes through plasmid vectors [16]; live vector vaccines, which utilize attenuated viruses or bacteria as efficient antigen delivery systems [17]; and nanoparticle vaccines, which significantly enhance antigen delivery efficiency and immunogenicity through specialized carriers [18]. The mode of immunization can be divided into active immunity and passive immunity. Active immunity occurs when the body itself encounters an antigen, producing antibodies and memory cells, and it takes effect slowly. Passive immunity involves the direct external acquisition of antibodies to rapidly neutralize pathogens or toxins, and it is suitable for emergency prevention or treatment of infectious diseases [19]. IgY antibodies are a type of passive immunity vaccine that obtain specific antibodies through the immunization of poultry. This not only avoids the risks associated with direct vaccination but also quickly establishes passive immune protection. Additionally, IgY has the advantages of high antigen specificity, low cost, high availability, simple preparation, no residue, and compliance with animal welfare regulations [20]. It provides an innovative solution for dealing with new drug-resistant pathogens and serves as a green and safe alternative to antibiotics [21].

Fish possess unique immune response characteristics, and their immune defense primarily relies on the non-specific immune system [22], including the skin mucus barrier and humoral factors such as the lysozyme, while their specific immune system is relatively simple, only capable of producing IgM-class antibodies and exhibiting a weak immune memory [23]. Although their well-developed mucosal immune system (especially in the gills and intestinal tissues) plays a crucial role in pathogen defense, the overall immune function is susceptible to environmental factors such as water temperature, displaying distinct temperature dependency and seasonal fluctuation characteristics [24,25]. As a passive immune vaccine, IgY antibodies can compensate for the deficiencies in the specific immunity of fish, enhance the immune function of antibodies, and promote the ability of fish to resist infections from various pathogens.

In this study, IgY antibodies against live *S. xiamenensis* (LSX-IgY) and inactivated *S. xiamenensis* (ISX-IgY) were prepared by immunizing laying hens. Through passive immunization of *Carassius auratus* with IgY antibodies and challenge experiments with *S. xiamenensis* and *A. hydrophila*, combined with an analysis of non-specific immune indicators, detection of protection rates, antioxidant and inflammatory response assays, and histopathological and immunofluorescence assessments of visceral tissues, a comprehensive evaluation was conducted on the bacterial resistance of LSX-IgY and ISX-IgY (Supplementary Figure S1). This study lays a theoretical foundation for the development of multivalent passive immunization vaccines for aquaculture.

## 2. Results

### 2.1. Passive Protection and Passive Cross-Protection Rates of IgY Antibodies in C. auratus

**Figure 1:**
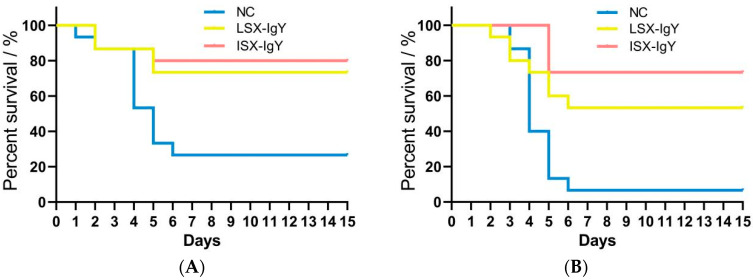
Survival rate of *C. auratus* infected with pathogenic bacteria. (**A**,**B**) represent infection with *S. xiamenensis* and *A. hydrophila*, respectively. NC, the nature control (blank IgY antibody).

Using the PEG6000 purification method, 2 mL of high-purity IgY antibodies of LSX-IgY and ISX-IgY was obtained and had no potential degradation (Supplementary Figure S2), each with a concentration of 1 μg/μL. To investigate the differences in the protective rates of LSX-IgY and ISX-IgY against bacterial infections in *C. auratus*, the two IgY antibodies were immunized goldfish and challenged to *S. xiamenensis* or *A. hydrophila*. The results showed that post-challenge, *C. auratus* exhibited symptoms such as slow swimming, epidermal hemorrhage, and abdominal swelling, accompanied by a significant number of deaths. The mortality rate stabilized after 6 days (Figure 1). The passive immunization protection rate of LSX-IgY against *S. xiamenensis* was 63.64% (*p* < 0.05), while that of ISX-IgY was 72.73% (*p* < 0.05). The passive cross-protection rate of LSX-IgY against *A. hydrophila* was 50% (*p* < 0.05), and that of ISX-IgY was 71.43% (*p* < 0.05) (Table 1). It can be concluded that both LSX-IgY and ISX-IgY exhibit significant immunization protection rates against infections by different bacterial species, with ISX-IgY showing better passive immunization protection effects, although the difference is not statistically significant.

### 2.2. Determination of Bacterial Counts in the Kidney of C. auratus

**Figure 2:**
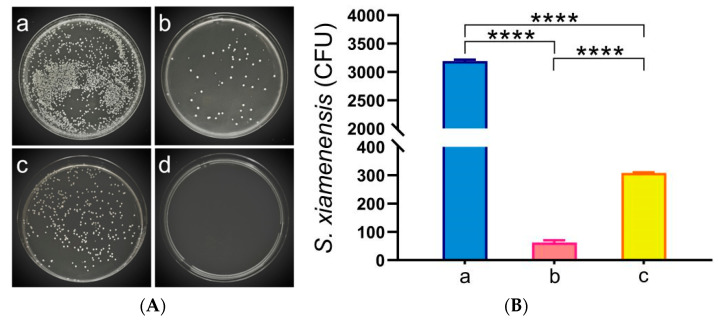

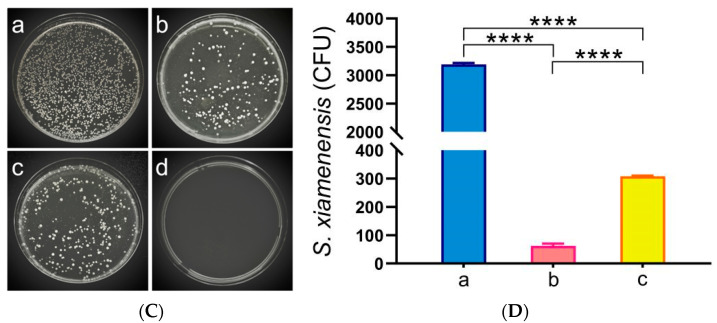
Kidney bacterial count statistics of *C. auratus*. (**A**) represents kidney bacterial colonies of *C. auratus* challenged with *S. xiamenensis*; (**B**) represents the bacterial colony count of *C. auratus* kidney challenged with *S. xiamenensis*; (**C**) represents kidney bacterial colonies of *C. auratus* challenged with *A. hydrophila*; (**D**) represents the bacterial colony count of *C. auratus* kidney challenged with *A. hydrophila*. a, b and c represent kidney bacterial colonies of *C. auratus* immunized with blank IgY antibodies as the nature control (NC), LSX-IgY, ISX-IgY, respectively. d represents an uninfected kidney (negative control). **** *p* < 0.0001.

To assess the changes in bacterial load within *C. auratus*, kidney tissues were sampled for smear culture on the second day post-challenge with *S. xiamenensis* and *A. hydrophila*. The results indicate that, compared to the NC, the bacterial counts in the kidneys of both the LSX-IgY and ISX-IgY were significantly reduced (*p* < 0.05) (Figure 2). This finding suggests that both LSX-IgY and ISX-IgY are effective in clearing bacteria from the kidneys, with LSX-IgY demonstrating a significantly better efficacy (*p* < 0.05).

### 2.3. Detection of Phagocytic Activity of Leucocytes in C. auratus

To evaluate the phagocytic activity of leucocytes, this study conducted phagocytosis assays using plasma after *C. auratus* immunized IgY antibody and challenged the pathogen. The results showed that both the LSX-IgY and ISX-IgY exhibited a significant increase in the phagocytic index (*PI*) and phagocytic rate (*PR*) of *C. auratus* leukocytes (*p* < 0.05) (Table 2). This indicates that both LSX-IgY and ISX-IgY can effectively activate the phagocytic activity of leukocytes, with no significant differences between the two IgY.

### 2.4. Detection of Antioxidant-Related Factors in the Serum of C. auratus

**Figure 3:**
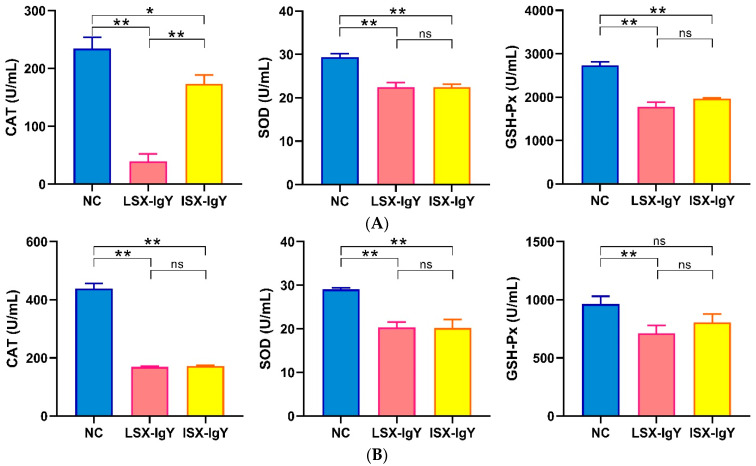
The expression levels of antioxidant-related factors. (**A**,**B**) represent *S. xiamenensis* and *A. hydrophila* challenge, respectively. * *p* < 0.05, ** *p* < 0.01. ns indicates no significant difference (*p* > 0.05). Compared to the NC group, the expressions of CAT, SOD, and GSH-Px decreased (*p* < 0.05) in LSX-IgY or ISX-IgY groups, with no significant differences between the two IgY.

To assess the levels of antioxidants in the serum of *C. auratus*, the antioxidant factors were evaluated in the serum on the second day after passive immunization with IgY antibodies and bacterial challenge. The results show that, compared to the control group, the levels of most antioxidant-related factors of superoxide dismutase (SOD), catalase (CAT), and glutathione peroxidase (GSH-Px) in the serum of the LSX-IgY and ISX-IgY groups were significantly reduced (*p* < 0.05) after challenge with *S. xiamenensis* or *A. hydrophila* (Figure 3). These results indicate that both LSX-IgY and ISX-IgY can significantly alleviate antioxidant effects in *C. auratus*, with no significant differences observed between the two types of IgY antibodies.

### 2.5. Detection of the mRNA Expression of Inflammation-Related Genes in C. auratus

**Figure 4:**
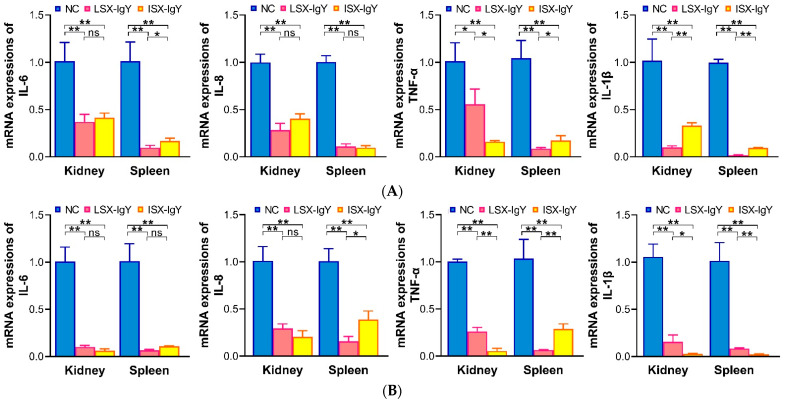
mRNA expression levels of inflammatory factors (IL-6, IL-8, TNF-α, and IL-1β) in internal organs. (**A**,**B**) represent challenges with *S. xiamenensis* and *A. hydrophila*, respectively. * *p* < 0.05, ** *p* < 0.01. ns indicates no significant difference (*p* > 0.05). Compared to the NC group, the mRNA expressions of IL-6, IL-8, TNF-α, and IL-1β decreased (*p* < 0.05) in LSX-IgY or ISX-IgY groups. The indicators of the LSX-IgY group were lower than that of the ISX-IgY group.

After passive immunization of *C. auratus* with IgY antibodies, the fish were challenged with *S. xiamenensis* or *A. hydrophila*, respectively. The mRNA expression levels of inflammation-related genes (*il-6*, *il-8*, *tnf-α*, and *il-1β*) in the kidney and spleen were evaluated. The results showed that compared to the NC, the mRNA expression levels of IL-6, IL-8, TNF-α, and IL-1β in the LSX-IgY and ISX-IgY groups were significantly reduced (*p* < 0.05) (Figure 4). The results indicate that both LSX-IgY and ISX-IgY can effectively alleviate the inflammatory response induced by *S. xiamenensis* and *A. hydrophila* in *C. auratus*.

### 2.6. The Interactions of IgY or C. auratus Serum with Pathogenic Bacteria In Vitro

**Figure 5:**
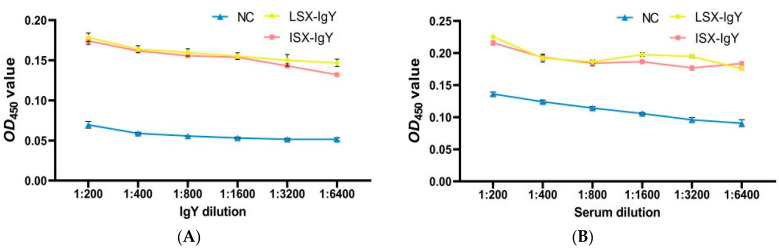

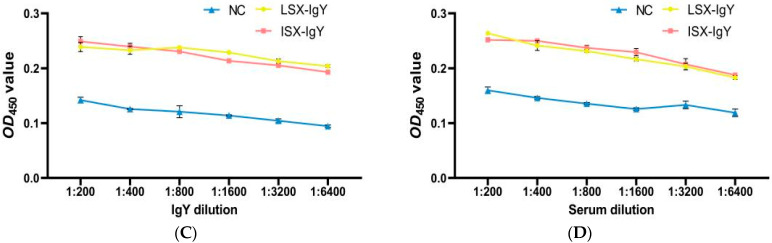
Immune recognition between the IgY or *C. auratus* serum and pathogens. (**A**) represents the recognition between IgY and *S. xiamenensis*. (**B**) represents the mutual recognition between the serum of *C. auratus* (immunized with IgY and challenged with *S. xiamenensis*) and *S. xiamenensis*. (**C**) represents the recognition between IgY and *A. hydrophila*; (**D**) represents the mutual recognition between the serum of *C. auratus* (immunized with IgY and challenged with *A. hydrophila*) and *A. hydrophila*.

To investigate the in vitro interaction between IgY antibodies and bacteria, an ELISA experiment was conducted. The results demonstrated that LSX-IgY and ISX-IgY could specifically bind to both *S. xiamenensis* and *A. hydrophila*, with the absorbance gradually decreasing with increasing antibody dilution. At a dilution of 1:6400, the two IgY antibodies still interacted with the pathogenic bacteria (*p* < 0.05) (Figure 5A,C).

Additionally, *C. auratus* was immunized with LSX-IgY or ISX-IgY and challenged with *S. xiamenensis* and *A. hydrophila*. The interaction between *C. auratus* serum and the pathogens was examined. The results showed that the absorbance decreased with increasing dilution of *C. auratus* serum; at a 1:6400 dilution, there was an interaction between the *C. auratus* serum and *S. xiamenensis* and *A. hydrophila*, in contrast to the NC (*p* < 0.05) (Figure 5B,D).

These results indicate that the LSX-IgY, ISX-IgY, and the sera of *C. auratus* immunized with LSX-IgY or ISX-IgY can effectively participate in antigen–antibody reactions. There is no significant difference between LSX-IgY and ISX-IgY.

### 2.7. Histopathological Observation of C. auratus Tissue Morphology

**Figure 6:**
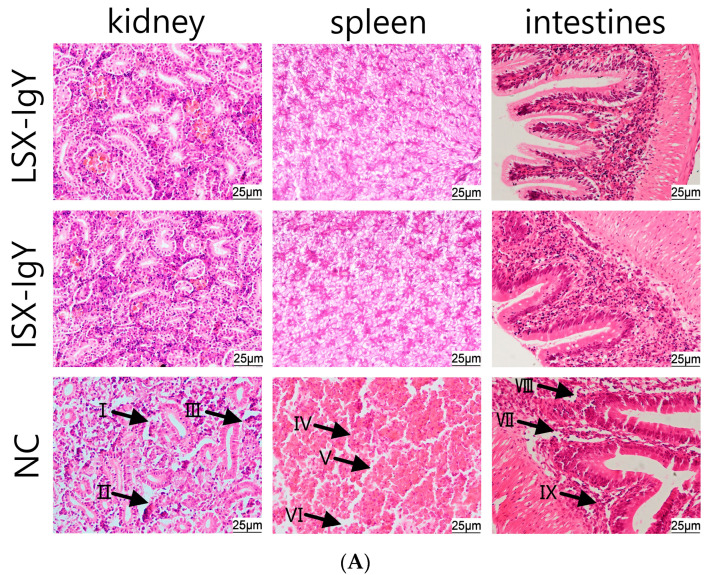

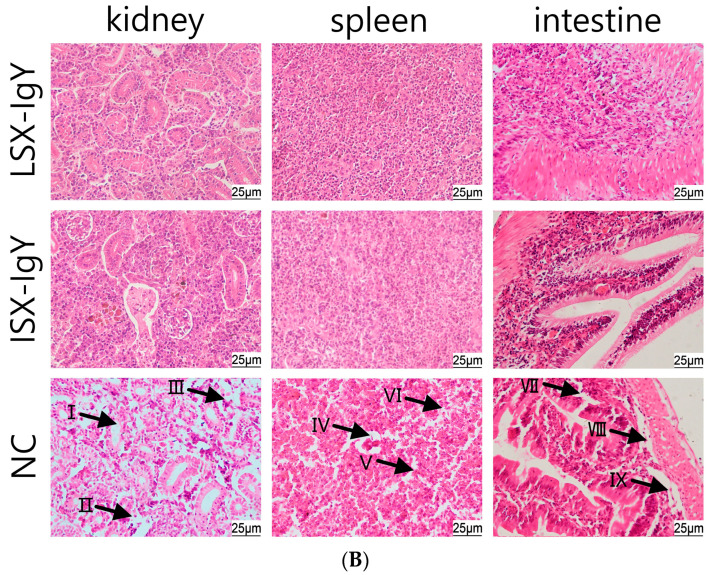
Histopathological sections of the kidney, spleen, and intestinal tissues of *C. auratus*. (**A**) represents infection with *S. xiamenensis*; (**B**) represents infection with *A. hydrophila*. (I) demonstrates loose renal tubular structure; (II) demonstrates glomerular atrophy; (III) demonstrates renal cell apoptosis; (IV) demonstrates low density of splenic cells; (V) demonstrates splenic cell apoptosis; (VI) demonstrates incomplete structure of splenic tissue; (VII) demonstrates intestinal villus necrosis; (VIII) demonstrate intestinal mucosal necrosis; (IX) demonstrates intestinal cell apoptosis.

To evaluate the organ damage in *C. auratus*, they were immunized with LSX-IgY and ISX-IgY and then we challenged them with *S. xiamenensis* or *A. hydrophila*. Kidney, spleen, and intestinal tissues were collected for histopathological examination. The results showed that the control group exhibited significant multi-organ pathological damage: glomerular atrophy, a loose renal tubular structure, and increased apoptosis were observed in kidney tissues; a sparse cell arrangement, structural disorder, and apoptotic body formation were present in spleen tissues; and thinning of the mucosal lamina propria, disruption of structural integrity, and aggregation of apoptotic cells were observed in intestinal tissues. In contrast, the kidneys, spleens, and intestinal tissue structures in both the LSX-IgY and ISX-IgY groups remained intact and clear, with no significant pathological damage observed (Figure 6). These results indicate that both LSX-IgY and ISX-IgY can effectively mitigate organ damage caused by pathogenic bacterial infection.

### 2.8. Immunofluorescence Analysis on Kidney Tissues of C. auratus

**Figure 7:**
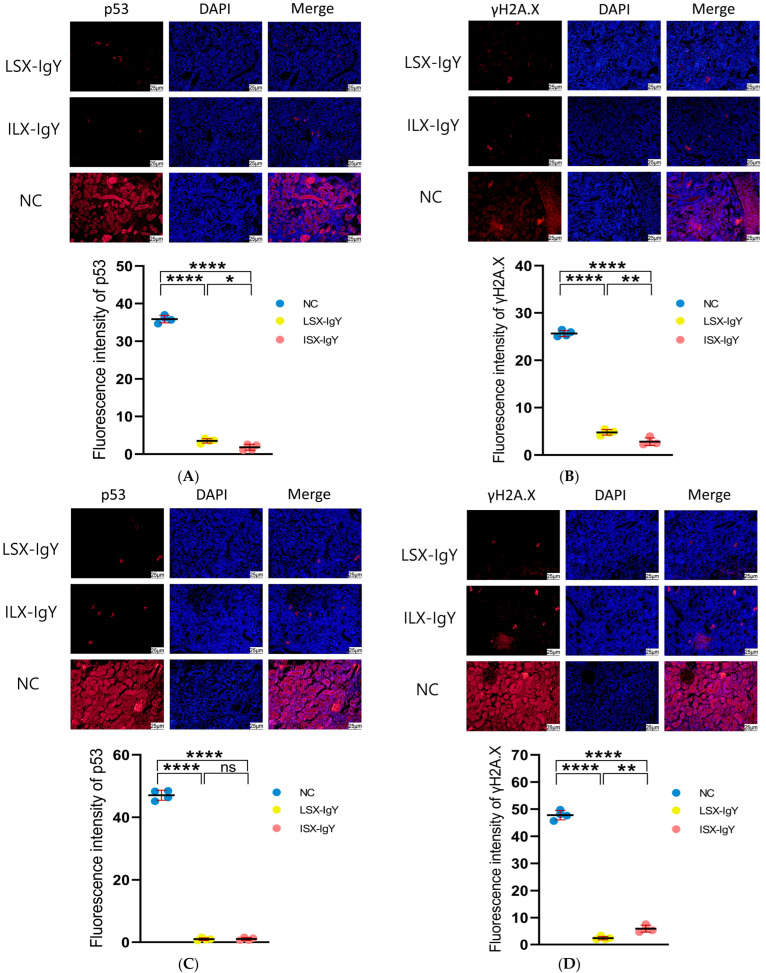
Immunofluorescence detection of p53 and γH2A.X proteins in the kidney of *C. auratus*. (**A**,**B**) represent the immunofluorescence of *C. auratus* kidneys infected with *S. xiamenensis*. (**C**,**D**) represent the immunofluorescence of *C. auratus* kidneys infected with *A. hydrophila*; (**A**,**C**) show the expression of p53. (**B**,**D**) show the expression of γH2A.X. ns indicates no significant difference. * *p* < 0.05, ** *p* < 0.01, **** *p* < 0.0001.

To evaluate the apoptosis of kidney cells, immunofluorescence analysis was conducted on the kidneys of *C. auratus*, with red fluorescence labeling proteins of p53 and γH2A.X and blue fluorescence labeling DAPI for nuclei. The results show that compared with the control, the expression levels of p53 and γH2A.X were significantly reduced in both the LSX-IgY and ISX-IgY immunization groups (*p* < 0.05) (Figure 7). The results indicate that passive immunization with LSX-IgY or ISX-IgY can effectively reduce apoptosis and DNA damage in the kidney cells of *C. auratus*.

## 3. Discussion

Polyvalent passive IgY vaccines have demonstrated unique advantages for aquaculture. They can broadly neutralize multiple drug-resistant pathogens, achieving cross-species protection from poultry to fish [26,27,28]. As IgY utilizes mature yolk antibody technology, it is low cost and convenient to use. They exhibit a rapid onset (2 h), are safe (no toxic side effects), and are environmentally friendly, making them particularly suitable for responding to sudden outbreaks and for large-scale prevention and control [29,30,31]. This study immunized laying hens with live or inactivated *S. xiamenensis*, prepared polyvalent passive IgY antibodies by collecting immunized eggs, and evaluated their immunoprotective effects against multiple pathogenic bacterial infections in *C. auratus*, providing a theoretical basis for the development of new vaccines in aquaculture.

The survival rate is a core indicator that directly reflects the protective efficacy of vaccines or therapeutic measures. By quantifying the proportion of surviving test organisms, the survival rate intuitively demonstrates the ability of an immune substance to resist lethal infections and provides critical evidence for efficacy evaluation and clinical application [32]. Zhou et al. infected suckling mice with HCoV-OC43 and treated them with arbidol (50, 25, and 12.5 mg/kg/d) via gavage. They found that arbidol increased the survival rate of suckling mice, indicating that it may be a useful method for the prevention and treatment of HCoV-OC43 [33]. Hu et al. treated a mouse model of acute methicillin-resistant *S. aureus* (MRSA) pneumonia with mFe-cinnamaldehyde + under ultrasonic (mFe-CA + US), significantly improving the survival rate of these mice. This unveiled an antibacterial alternative that induces ferroptosis in MRSA, providing a target and a theoretical basis for the clinical treatment of acute MRSA pneumonia [34]. Zhao et al. treated EV71-infected mice with honokiol, significantly enhancing their survival rate, revealing that honokiol may be a candidate drug for the development of anti-EV71 drugs [35]. In this study, we also immunized *C. auratus* with live and inactivated bacterial IgY antibodies and found that the passive immunization protection rate of LSX-IgY against *S. xiamenensis* was 63.64% (*p* < 0.05), while that of ISX-IgY was 72.73% (*p* < 0.05), and the passive cross-protection rate of LSX-IgY against *A. hydrophila* was 50% (*p* < 0.05), and that of ISX-IgY was 71.43% (*p* < 0.05). In the preliminary phase, we conducted an experiment on the treatment of bacterial infections in *C. auratus* using the antibiotic Enrofloxacin. Briefly, *C. auratus* was intraperitoneally injected with *S. xiamenensis* (1 × 10^7^ CFU) or *A. hydrophila* (1 × 10^7^ CFU) and treated with different concentrations of Enrofloxacin via feeding. The results indicated that if the immune protection rate reached 65% (corresponding to this study), the Enrofloxacin treatment concentration should not be less than 8 μg/g. It is evident that the 2 μg/g IgY antibody is equivalent in effect to the 8 μg/g Enrofloxacin in treating bacterial infections in *C. auratus*. These results confirm that IgY antibodies exhibit immunization protection effects against different bacterial species, and there was no significant difference in these effects between LSX-IgY and ISX-IgY.

Non-specific immunity serves as the first line of defense against pathogen invasion in organisms [36]. In aquatic animals, due to the relatively underdeveloped specific immune system, non-specific immunity plays a dominant role [37]. Common evaluation indicators for non-specific immunity include cell phagocytosis, bacterial counts in visceral infections, in vitro antigen–antibody interaction assays, and immune factor detection. Li et al. found that a carnivorous diet significantly enhances the antioxidant capacity and non-specific immunity of hybrid groupers [38]. Ding et al. treated *Megalobrama amblycephala* with mannan oligosaccharides (MOSs), which increased the activity of hepatic antimicrobial and antioxidant enzymes, thereby enhancing the host’s bactericidal and antioxidant capabilities [39]. Lu et al. employed copper as a signaling molecule in a zebrafish model, demonstrating enhanced recruitment of phagocytic cells and promoting bacterial clearance [40], while Zhang et al., in their study of *Streptococcus equi*, found that the srtA-5012 (R147G) mutant exhibited the lowest bacterial load in the lungs [41]. Oostindie et al., through in vitro antigen–antibody interaction assays, showed that effective antibodies can neutralize invading pathogens or pathogenic molecules by binding interference and mediating humoral and cellular effector functions, offering promising therapeutic options for a variety of diseases [42]. In this study, *C. auratus* was immunized with live and inactivated bacterial IgY antibodies and challenged to bacteria, and the phagocytic activity of leukocytes from goldfish significantly increased. Meanwhile, the bacterial load in the kidneys of *C. auratus* was significantly reduced. Additionally, both the two IgY antibodies and the *C. auratus* serum could specifically bind to the pathogens (*p* < 0.05). Thus, this study systematically evaluated the non-specific immune activity of IgY. However, due to the bacterial challenge experiment being conducted 2 h after the immunization of *C. auratus* with IgY, the relatively short duration of action resulted in the immune factors not yet being produced, making them unmeasurable. These results indicate that LSX-IgY and ISX-IgY activated the non-specific immunity of *C. auratus*.

The antioxidant factors in fish serum, such as superoxide dismutase (SOD), catalase (CAT), and glutathione (GSH-Px), are key molecules that maintain the intracellular redox balance. They can eliminate excessive reactive oxygen species (ROS) and thereby protect cells from oxidative damage [43]. Huo et al. found that feeding mandarin fish (*Siniperca chuatsi*) with CI20 effectively enhanced their antioxidant capacity and immunity [44]. Shi et al. also found that antioxidant indicators (SOD and CAT) and innate immune parameters (LZM, C3) were downregulated in untreated fish under normal conditions [45]. Meanwhile, Vieira et al. showed that the expression of glutathione peroxidase (GSH-Px), catalase (CAT), superoxide dismutase (SOD), and lipid peroxidation markers (LPO) was downregulated in Amazonian fish not exposed to mercury compared to those exposed to mercury [46]. In this study, the levels of antioxidant factors (such as SOD, CAT, and GSH-Px) in the serum of *C. auratus* were significantly reduced after immunizing LSX-IgY or ISX-IgY and challenging bacteria. The expression of these antioxidant factors reflects the antioxidant status within the fish body. This study demonstrated that the IgY reduced oxidative damage in fish after exposure to pathogenic bacteria, which is consistent with previous findings on the antioxidant effects of immunoreactive substances. Therefore, the two IgY antibodies can alleviate the oxidative stress response induced by pathogenic bacterial infection.

The expression levels of inflammation-related genes (such as IL-6, IL-8, TNF-α, and IL-1β) are important indicators for assessing the intensity of the inflammatory response [47]. Fu et al. discovered that NOD-like receptor family pyrin domain-containing 3 (NLRP3) triggers the upregulation of cytokines IL-1β and IL-18, which activates inflammatory expression [48]. Yu et al. found that Soc-miR-118 directly targets IL-6 to downregulate and inhibit the production of inflammatory cytokines, thereby preventing excessive inflammatory responses [49]. Chen et al. demonstrated that feeding inflammatory bowel diseased (IBD) mice with palmitoleic acid could reduce inflammatory cell infiltration as well as the expression of TNF-α and IL-6 [50]. In this experiment, the expression of inflammation-related genes (such as IL-6, IL-8, TNF-α, and IL-1β) in the kidneys and spleens of *C. auratus* immunized with LSX-IgY or ISX-IgY was significantly downregulated. Meanwhile, these inflammation-related factors reflect the inflammatory state of the organism, with low expression indicating a reduction in inflammation [48,49]. Thus, this study demonstrated that fish immunized with IgY reduced the inflammatory response following bacterial infection in fish. These results indicated that the two IgY antibodies could suppress the excessive inflammatory response induced by pathogenic bacterial infection, and IgY had an anti-inflammatory effect.

Histopathology evaluates the extent of damage caused by pathogenic infections to host organs by observing changes in tissue structure and cell morphology. Immunofluorescence technology can detect the degree of cell apoptosis and DNA damage through labeling specific proteins such as p53 and γH2A.X [51]. Histopathological sections of the kidney, spleen, and intestine can visually reflect the pathological changes in these organs during infection. Schiffer et al. administered a NADPH oxidases (NOX4) inhibitor to the mice with acute kidney injury and assessed the integrity of the renal tissue structure and effect through histopathology, finding that the renal structure was more intact in the group treated with the NOX4 inhibitor [52]. Zhou et al. established a mouse xenograft tumor model using SCC7 cells and injected heat-inactivated *Prevotella intermedia* into the tumor tissue and observed the growth of the transplanted tumor in mice through histopathology [53]. Additionally, Lou et al. discovered that the binding of CDR1as to p53 disrupts the p53/MDM2 complex to prevent p53 ubiquitination and degradation, revealing that p53 expression is upregulated when DNA damage is irreparable via immunofluorescence technology [54]. Meanwhile, Arnould et al. demonstrated that γH2A.X detection can help assess whether pathogens induce genomic instability, thereby elucidating their pathogenic mechanisms [55]. In this study, histopathological analysis revealed that the structures of the kidney, spleen, and intestinal tissues of *C. auratus* treated with LSX-IgY or ISX-IgY remained intact without significant pathological damage. Meanwhile, the integrity of visceral tissues is crucial for the functionality of the fish organism [52], and the two IgY immunizations can protect the fish organism against bacterial infections. Further, in this study, the mRNA expression levels of γH2A.X and p53 in the kidney cells of IgY-treated *C. auratus* were significantly decreased. In addition, P53 and γH2A.X are important indicator proteins of cell apoptosis and DNA damage, respectively. Thus, the expression of P53 and γH2A.X can indirectly evaluate the immunoprotective effects of drugs on cell function [51], and the two IgY immunizations can reduce the apoptosis and DNA damage for visceral tissue cells. These results confirm the role of LSX-IgY or ISX-IgY antibodies in protecting the integrity of the host’s visceral tissues.

DNA and subunit vaccines belong to active immunization vaccines, and they stimulate the body to produce humoral and cellular immunity through animal immunization, requiring an induction period of 1 to 4 weeks. For explosive pathogenic bacterial infections, the immune effects of DNA and subunit vaccines are insufficient. DNA vaccines involve the direct injection of a recombinant eukaryotic expression vector that encodes a specific protein antigen into an animal’s body and utilize the animal’s cellular expression system to synthesize antigen proteins, thereby activating the immune response [56]. Subunit vaccines involve immunizing animals with specific antigenic fragments of the pathogen (proteins or polysaccharides), thereby activating the immune response [57]. IgY antibodies belong to passive immunization vaccines. Being immunized with IgY enables the animals’ organism to acquire immunity immediately, providing protection against explosive pathogenic bacterial infections, showing application value in aquaculture. Further, *C. auratus* belongs to the genus *Carassius* of family Cyprinidae, exhibiting strong environmental adaptability and possessing value for food, appreciation, and scientific research. Additionally, the genome and proteome sequences of *C. auratus* are relatively clear, so they are frequently used in the evaluation of drug activity in aquaculture [27]. Therefore, this study employs *C. auratus* as the evaluation animal for IgY immune ability.

In aquaculture, oral immunization is simple to administer and can be delivered through feed or bait, and it is relatively safe and does not require specialized equipment or technical personnel to save costs [26,35]. These advantages make oral immunization a highly promising method for vaccine delivery in fish, especially considering the economic benefits and practical applicability in large-scale aquaculture. However, the animal digestive system contains digestive fluids that have a degrading effect on vaccines, and mucosal absorption efficiency also needs further verification. Additionally, the current study only utilized intraperitoneal injection for IgY antibodies, which increased the cost of immunization in aquaculture. Meanwhile, intraperitoneal immunization cannot easily avoid the instability in digestive fluids or lack of mucosal absorption in fish. Further, this study was conducted entirely under laboratory conditions. For a claim of practical application in aquaculture, testing under field or semi-field conditions can better reflect the large-scale application effect of IgY vaccines. Therefore, it is necessary to carry out the assessment of the oral immunization approach, the instability, mucosal absorption efficacy, and scale testing for IgY vaccines in a future study.

In summary, IgY antibodies of LSX-IgY and ISX-IgY were prepared by immunizing laying hens. The two IgY antibodies have passive immune abilities to resist *S. xiamenensis* or *A. hydrophila* infections in *C. auratus*, and there is no significant difference between the two. LSX-IgY and ISX-IgY have the potential to serve as passive immunization vaccines in aquaculture. Further, the ISX-IgY obtained from immunizing laying hens with formaldehyde-inactivated *S. xiamenensis* causes minimal harm to the hens’ bodies, aligning with animal welfare standards; meanwhile, it has little impact on the hens’ growth and egg production. Therefore, ISX-IgY is expected to serve as a multivalent vaccine against major aquaculture pathogens.

## 4. Materials and Methods

### 4.1. Bacterial Strains and Animals

The Microbiology Laboratory of Fuyang Normal University in Anhui possesses preserved strains such as *S. xiamenensis*, *A. hydrophila*, and *Staphylococcus aureus*. Twenty-week-old Laihong laying hens were purchased from Chongqing Tengxin Biotechnology Co., Ltd. (Chongqing, China), and *C. auratus* (20 ± 1.0 g) was obtained from the Aquaculture Center of Fuyang City, Anhui province (Fuyang, China). All animal experiments were conducted in accordance with the “Guide for the Care and Use of Laboratory Animals” and approved by the Institutional Animal Care and Use Committee of Fuyang Normal University, China (No: 2024-04-005).

### 4.2. Preparation of IgY Antibodies

*S. xiamenensis* was inoculated into LB medium, and the bacterial cells were collected at an *OD*_600_ of 1.0. A portion of the collected cells was fully suspended in a 1% formaldehyde solution and inactivated by placing it in an 80 °C water bath for 90 min. The remaining portion was prepared for live bacterial immunization. A total of 400 μL of both live and inactivated *S. xiamenensis* (2 × 10^7^ CFU) was intramuscularly injected multiple times into laying hens, respectively. Immunization was performed four times, with an interval of 14 days between each immunization. Eggs were collected 7 days after the fourth immunization and stored at 4 °C in the dark. Using an egg yolk separator to separate the yolk from the egg, phosphate-buffered saline (PBS) solution (pH 7.2) was added in a volume of twice that of the yolk and mixed thoroughly. Then, polyethylene glycol (PEG6000) (Sangon Biotechnology Co., Ltd., Shanghai, China) was added with stirring to achieve a final concentration of 3.5% and then mixed further. The mixture was transferred into a 50 mL centrifuge tube, centrifuged at 10,000 r/min at 4 °C for 20 min, and the clear liquid was filtered into a beaker using filter paper, discarding the precipitate. The clear liquid was continuously stirred while gradually adding PEG6000 to achieve a final concentration of 8.5%. The solution was transferred into a 50 mL centrifuge tube, placed in a shaker at 25 °C with a speed of 100 r/min, and shaken for 30 min. After being left to stand for 10 min, it was centrifuged for 20 min. The supernatant was discarded, and the precipitate was thoroughly dissolved in 10 mL of phosphate-buffered saline (PBS) with continuous stirring, while PEG6000 was added to a final concentration of 12%. After complete dissolution, the above steps were repeated. The precipitate was centrifuged and dissolved in 2 mL of PBS solution. The solution was transferred into a dialysis bag and dialyzed in PBS solution at 4 °C for 36 h. The dialysate was the IgY antibody solution. Then, the concentration of IgY antibody was adjusted to 1 μg/μL with a bicinchoninic acid (BCA) protein assay kit (Sangon Biotechnology Co., Ltd., Shanghai, China) and stored at −80 °C in a refrigerator [26].

### 4.3. The Detection of Interactions of IgY or C. auratus Serum with Pathogenic Bacteria In Vitro

*S. xiamenensis* and *A. hydrophila* were inoculated into LB medium, and the bacterial cells were collected when the *OD*_600_ reached 1.0. The bacterial cells with a dose of 200 μL (2 × 10^7^ CFU) were added to the wells of an ELISA plate. The bacterial solution was placed at 4 °C overnight for coating. After coating was completed, the wells were washed with a phosphate-buffered saline with Tween (PBST) solution. Then, a 5% skim milk solution was added to the wells for blocking, which was then typically incubated at 37 °C for 1.5 h. After blocking was completed, another round of washing was performed. Next, the *C. auratus* serum (immunized with IgY and challenged to bacteria) or IgY antibodies were diluted (dilution ratios of 1:200, 1:400, 1:800, 1:1600, 1:3200, 1:6400), respectively, and added to each well for incubation. Incubation was carried out at 37 °C for 2 h. After washing, the secondary antibody (dilution ratio of 1:1000) (Sigma-Aldrich, St. Louis, MO, USA) was added and incubated at 37 °C for 1.5 h. After the incubation was completed, the wells were washed again with PBS solution. Subsequently, the chromogenic solution was added, typically incubated at 37 °C for 10 min, and the reaction was terminated by adding the stop solution. The reaction results were read using a microplate reader at a wavelength of *OD*_450_ nm [27].

### 4.4. Passive Protection and Passive Cross-Protection of IgY Antibodies

In the experiment, the *C. auratus* was divided into 6 groups, with 15 fish in each group. The specific grouping was as follows: three groups were challenged with *S. xiamenensis*, including the blank IgY group as the nature control group (NC), the live bacteria IgY antibody group (LSX-IgY), and the inactivated bacteria IgY antibody group (ISX-IgY), and the other three groups were challenged with *A. hydrophila* in the same manner. Each fish was first intraperitoneally injected with a dose of 40 μL of IgY antibodies (40 μg). Two hours later, the fish were challenged with *S. xiamenensis* (1.0 × 10^9^ CFU) or *A. hydrophila* (4.3 × 10^9^ CFU), respectively. Following the challenge, mortality was observed for 14 days. The relative percent survival (RPS) was computed using the following formula: RPS (%) = (1 − [Experimental group mortality%/Control group mortality%]) × 100. Experimental group mortality refers to the mortality rate after administering LSX-IgY and ISX-IgY, while control group mortality refers to the mortality rate of the blank IgY group. All data were statistically analyzed using SPSS 19.0 software [26].

### 4.5. Renal Bacterial Count

*C. auratus* was immunized with IgY antibodies and challenged with *S. xiamenensis* or *A. hydrophila*, while the control group was injected with blank egg IgY antibodies. Two days later, kidney tissues were collected under anesthesia and homogenized under aseptic conditions using 400 μL of PBS, and it was repeated three times. To count bacterial colonies, the kidney homogenate was evenly spread on the surface of LB medium and incubated overnight at 37 °C in a constant-temperature incubator. After incubation, the bacterial colonies on the medium were observed and counted, and the total number of colonies was calculated to evaluate the inhibitory effect of IgY antibody immunization on bacterial infections [27].

### 4.6. Analysis of the Leukocyte Phagocytic Activity

First, the *C. auratus* was challenged with *S. xiamenensis* or *A. hydrophila* after immunizing to IgY antibodies. Two days later, blood plasma was collected via the tail vein using anticoagulant centrifuge tubes under anesthesia, and it was repeated three times. A total of 0.2 mL of plasma was mixed with *S. aureus* (2 × 10^6^ CFU) that was inactivated with 1% formaldehyde. The mixture was incubated in a water bath at 25 °C for 60 min. After incubation, the mixture of plasma and inactivated bacteria was added dropwise onto a glass slide and evenly spread to create a blood smear. The blood smear was stained using a Rapid Giemsa Staining Kit (Sangon Biotech Co., Ltd., Shanghai, China). After staining was conducted according to the kit’s operating instructions, the smear was observed under a microscope to count phagocytic cells. The calculation methods are as follows: phagocytic rate (*PR*%) = number of cells involved in phagocytosis among 100 phagocytic cells/100 × 100% and phagocytic index (*PI*) = number of bacteria in phagocytic cells/number of cells involved in phagocytosis [27].

### 4.7. Analysis of Antioxidant Factors

After being challenged with *S. xiamenensis* and *A. hydrophila*, serum was obtained from the caudal vein of the *C. auratus* under anesthesia, and it was repeated three times. The levels of antioxidant factors, including superoxide dismutase (SOD), catalase (CAT), and glutathione peroxidase (GSH-Px), were assessed according to the instructions of the detection kits (Sangon Biotechnology Co., Ltd., Shanghai, China) [58].

### 4.8. Inflammatory Factor mRNA Expression

The expression of inflammatory factor mRNA was assessed using real-time quantitative PCR. In brief, on the second day after bacterial challenge, the kidneys and spleens of the *C. auratus* were collected under anesthesia, and the tissues were thoroughly ground in liquid nitrogen to extract RNA according to the instructions of the RNA extraction kits (TAKARA, Tokyo, Japan), which was then converted to cDNA according to the instructions of the kit (Takara, Beijing, China), and it was repeated three times. Real-time quantitative reverse-transcription PCR (qRT-PCR) was performed using an SYBR^®^ Green Premix kit (Takara, Beijing, China) and primers were synthesized (Supplementary Table S1). Simply put, the ΔCt (cycle threshold change) was obtained by comparing the Ct value of the target gene with that of the reference gene of glyceraldehyde-3-phosphate dehydrogenase (*gapdh*). Then, ΔΔCt was derived by comparing the ΔCt values between the experimental group and the control group. Finally, the mRNA expression was analyzed using the 2^−ΔΔCt^ method [26].

### 4.9. Tissue Pathological Analysis

After challenge with bacteria, kidney, spleen, and intestine tissues of *C. auratus* were collected and immersed in Davidson’s fixative and a 10% formalin solution for 24 h for fixation, and it was repeated three times. Following fixation, the tissues were dehydrated through a graded ethanol series (70%, 80%, 90%, 95%, and 100% ethanol), then cleared with xylene twice, each for 30 min. The cleared tissues were embedded in paraffin at 60 °C and sectioned into 4 μm thick slices using a paraffin microtome (Leica, Wetzlar, Germany). The sections were placed on glass slides and dried on a slide warmer at 60 °C for 3 h. Subsequently, the tissue sections were deparaffinized with xylene, dehydrated through a graded ethanol series, and cleared with xylene again, followed by staining with hematoxylin and eosin (H&E). After staining, the sections were cleared with xylene, mounted with neutral resin, and observed and photographed under an optical microscope (Leica, Wetzlar, Germany) [59].

### 4.10. Renal Immunofluorescence Analysis

The prepared kidney tissue sections were placed in xylene for dewaxing, followed by hydration in a descending ethanol concentration gradient, and it was repeated four times. After treatment with an antigen retrieval solution, an immunohistochemical pen was used to draw a circle around the tissue, and 50 μL (5%) bovine serum albumin (BSA) blocking solution was added within the circle to facilitate blocking at room temperature for 1.5 h. After blocking, the slides were washed with PBST, monoclonal antibodies against p53 or γH2A.X (dilution ratio 1:500) (Sigma-Aldrich, St. Louis, MO, USA) were added to the tissue, and the slides were incubated overnight at 4 °C. After washing, the secondary antibody solution (dilution ratio 1:1000) was added and incubated at 37 °C for 1 h. After washing, the nuclei were stained with 4′,6-diamidino-2-phenylindole (DAPI) at room temperature in the dark for 10 min, the kidney tissue sections were mounted, and photographs were taken under a fluorescence microscope [27].

### 4.11. Statistical Analysis

All the experimental data were expressed as mean ± SD, and all experiments were repeated at least three times. The significant difference from the respective control in all experiments was assessed via one-way analysis of variance (ANOVA) using SPSS (IBM Corporation, Chicago, IL, USA). Values of *p* < 0.05 were considered statistically significant [60].

## 5. Conclusions

This study prepared LSX-IgY and ISX-IgY and evaluated their immunoprotective effects through passive administration of *C. auratus* and challenge by *S. xiamenensis* and *A. hydrophila*. The results indicated that LSX-IgY or ISX-IgY exhibited immune-protective rates, reduced the bacterial load in the kidney, and enhanced the phagocytic activity of leukocytes. Meanwhile, the two IgY reduced the levels of antioxidant factors and the mRNA expression of inflammation-related genes. Additionally, the two IgY, and the sera of *C. auratus,* could recognize the bacteria in vitro. Further, the two IgY could maintain the structural integrity of visceral tissues and decreased apoptosis and DNA damage in kidney cells. Therefore, LSX-IgY and ISX-IgY demonstrate efficacy against multiple bacterial infections, with no significant differences between the two. Considering that inactivated bacterial immunization aligns more closely with welfare standards for laying hens, ISX-IgY is anticipated as a polyvalent vaccine candidate for multiple bacterial infections in aquaculture.

## Figures and Tables

**Table 1:** Passive protection and passive cross-protection rates of IgY antibodies in *C. auratus*.

Bacterium	IgY Antibody	No.	Survival, No.	Death, No.	ADR, %	RPS, %	RPS, *p*-Value
*S. xiamenensis*	NC	15	4	11	73.34	--	--
	LSX-IgY	15	11	4	26.67	63.64 **	0.011
	ISX-IgY	15	12	3	20.00	72.73 **	0.003
*A. hydrophila*	NC	15	1	14	93.33	--	--
	LSX-IgY	15	8	7	46.67	50.00 *	0.005
	ISX-IgY	15	11	4	26.67	71.43 **	0.002

Notes: ADR, accumulating death rate. RPS, the relative percent survival. RPS (%) = 1 − (% vaccinated mortality/% non-vaccinated mortality) × 100. NC, the nature control (blank IgY antibody). * *p* < 0.05, ** *p* < 0.01 (compared with control).

**Table 2:** Results of the phagocytosis experiment on pathogenic bacteria in *C. auratus*.

Bacterium	Groups	*PR*%	*PI*	*PR*% (*p*-Value)	*PI* (*p*-Value)
*S. xiamenensis*	NC	36.67 ± 5.69	0.69 ± 0.09	--	--
	LSX-IgY	70.56 ± 5.27 **	1.51 ± 0.04 **	0.0017	0.0001
	ISX-IgY	60.47 ± 3.25 **	1.43 ± 0.05 **	0.0035	0.0002
*A. hydrophila*	NC	35.28 ± 3.62	0.81 ± 0.04	--	--
	LSX-IgY	50.34 ± 4.67 *	1.51 ± 0.15 **	0.0116	0.0016
	ISX-IgY	49.67 ± 8.36 *	1.45 ± 0.08 **	0.034	0.0003

Notes: * *p* < 0.05, ** *p* < 0.01 indicate significant differences between the LSX-IgY or ISX-IgY group and the NC group in the two types of challenge bacteria, respectively. The calculation methods are as follows: phagocytic rate (*PR*%) = number of cells involved in phagocytosis among 100 phagocytic cells/100 × 100% and phagocytic index (*PI*) = number of bacteria in phagocytic cells/number of cells involved in phagocytosis.

## Data Availability

The data presented in this study are available on request from the corresponding author.

## Supplementary Materials

The following supporting information can be downloaded at: https://www.mdpi.com/article/10.3390/ijms26147012/s1.
