# Supplementary material for: Multivalent Immune-Protective Effects of Egg Yolk Immunoglobulin Y (IgY) Derived from Live or Inactivated Shewanella xiamenensis Against Major Aquaculture Pathogens
Source: Int J Mol Sci. 2025 Jul 21;26(14):7012. doi: 10.3390/ijms26147012 (PMC12295794; doi:10.3390/ijms26147012)
Supplement: Supplementary file 1 [file ijms-26-07012-s001.zip › Supplementary Table S1.pdf]

**Supplementary Table S1.** Primers used for the qRT-PCR.

| Gene | NCBI number | Forward primer (5–3′) | Reverse primer (5–3′) |
|---|---|---|---|
| *il-6* | XM_026289280.1 | TCTCCTCAGACCCTCAGACG | CGTTTGGTCCCGTGTTTGAC |
| *il-8* | XM_026267284.1 | GGAGTGCAGGCCACTGTTAG | ATCAGAAGCATGAAGGCGGA |
| *il-1β* | AJ249136.1 | TTCAGGAAAGAGACGGGCAC | GTCAGTTGGCACCTGGATCA |
| *tnf-α* | EU069817.1 | GGGCCACATCGTGATTCGTA | GCCTCCAGTGTAGCATGTGT |
| *gapdh* | XM_026284269.1 | GATTTCAACGGGGATGTGCG | TCACACACACGGTTGCTGTA |
