# Supplementary figures and images for: Multivalent Immune-Protective Effects of Egg Yolk Immunoglobulin Y (IgY) Derived from Live or Inactivated Shewanella xiamenensis Against Major Aquaculture Pathogens
Source: Int J Mol Sci. 2025 Jul 21;26(14):7012. doi: 10.3390/ijms26147012 (PMC12295794; doi:10.3390/ijms26147012)

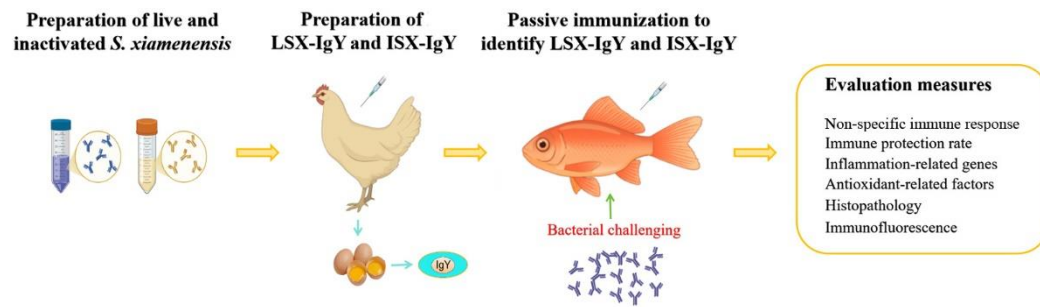

**Supplementary Figure S1.** Experimental process.

Supplement: Supplementary file 1 [file ijms-26-07012-s001.zip › Supplementary Figure S1.pdf]
